# Resistance to Cyclic Fatigue of Nickel-Titanium Files Immersed in Sodium Hypochlorite at Body Temperature

**DOI:** 10.1155/2020/8830163

**Published:** 2020-09-19

**Authors:** Hind F. Abuhulaibah, Ammar AbuMostafa

**Affiliations:** Restorative Dentistry Department, Endodontics Division, Riyadh Elm University, Riyadh, Saudi Arabia

## Abstract

**Materials and Methods:**

Forty-five files from each brand were randomly assigned to three groups (*n* = 15) and subjected to the following: no immersion (control), 1-minute immersion, and 5-minute immersion in 2.5% NaOCl at 37°C. CF for all the files was tested within a well-lubricated stainless-steel artificial canal in a water bath at 37°C simulating body temperature. The procedure was video recorded, and the number of cycles to fracture (NCF) was calculated by multiplying the time taken to fracture, with the number of rotations per second. The data were analyzed for normality, and thereafter, one-way ANOVA with multiple Bonferroni was used as the post hoc test.

**Results:**

The resistance to CF of OC files was significantly higher than PTG files in all groups. In groups immersed in NaOCl for one minute, OC files showed significant drop in the CF resistance; PTG files showed a drop as well but not significantly. Both files demonstrated a significant drop in resistance to CF after immersion in NaOCl for five minutes compared to the control group.

**Conclusion:**

OC files were significantly more resistant to CF compared to PTG in all groups. Immersion in 2.5% NaOCl for 5 minutes significantly reduced the resistance to CF for all the files.

## 1. Introduction

Present day nickel-titanium (NiTi) rotary files are used for root canal cleaning and shaping mainly because of their superelasticity [1]. NiTi files were introduced during the 1980s, which offered significant advantages such as the ability to maintain the original root canal shape and prevent the creation of irregularities inside the root canal [2].

Despite these advantages, NiTi files appear to have a risk of separation mainly because of flexural cyclic fatigue and torsional fracture [3]. A study demonstrated that 70% of fractures were because of cyclic fatigue, while 30% was torsional fatigue [4]. Researchers in another study evaluated 27 fractured ProTaper files and found flexural fatigue to be the major reason for failure (92.5%) [5]. Cyclic fatigue failure happens as a result of tension-compression stress cycles in the area of maximum flexure, particularly during preparing curved canals. Torsional fracture occurs when part of the file is locked to the dentin, while the shank maintains to rotate [6].

Corrosion is an additional factor that might limit the resistance to fatigue fracture, and it occurs during root canal cleaning and shaping when files come in contact with sodium hypochlorite (NaOCl) solution. Pits and surface defects are created which act as propagating point for fatigue failure of the files [3]. Effect of NaOCl on the cyclic fatigue resistance of the NiTi files was investigated through several studies, and findings were controversial [7–13]. Moreover, size, cross section, and core area of the files affect their cyclic fatigue resistance [14, 15].

The technology of the manufacture of the NiTi instruments is always being improved. The aim of the improvement is to get the instruments with superior flexibility and better resistance versus cyclic fatigue; not only the development of the manufacturing method but also the usage of the new alloys helps to increase the effectiveness and safety of the NiTi instruments. Improvement of the cross-sectional design, thermal treatment protocols, electropolishing, and electrodischarge machining (EDM) are some of the preferred methods [16].

ProTaper Gold files (Dentsply Sirona, York, PA) (PTG) were commercially introduced in 2014, geometrically identical to the ProTaper Universal (Dentsply Sirona, York, PA) design, but with enhanced metallurgical properties owing to the heat treatment process of the alloy [17]. It has been demonstrated that the fatigue resistance of ProTaper Gold was higher than ProTaper Universal [18].

Later, in 2018, One Curve files (Micro Mega, Besancon, France) (OC) were produced using a heat-treated C-wire technology. According to the manufacturer, the file has a controlled memory, which can present a better performance in the root canal along with enhanced accessibility [19]. Several studies have investigated the cyclic fatigue resistance of OC files, all of which demonstrated a high resistance to fracture. [20–24].

A literature search revealed that no prior research has evaluated the effects of immersion in sodium hypochlorite on the cyclic fatigue resistance of OC in comparison with PTG. The aim of the study was to assess the resistance to cyclic fatigue of the two nickel-titanium (NiTi) files, One Curve and ProTaper Gold, after immersion in NaOCl solution (2.5%) at body temperature for varying time intervals. The null hypothesis was that there would be no difference in the cyclic fatigue resistance of the tested files.

## 2. Materials and Methods

Two commercially available NiTi rotary files, namely, OC and PTG, with equal sample distribution (*n* = 45), were used in this study. All the files tested possessed an ISO tip size of 25 and length of 25 mm and were examined for defects or deformities prior to the experiment, using an operating microscope under ×25.6 magnification (Zumax OMS2350, Dental Microscope); no file was discarded. The 45 files of the same brand were randomly distributed into three groups of 15 each. The first group (control) composed of new files that were not immersed in solution. All files in the second and third groups were immersed in NaOCl 2.5% (Diaa, USA) at 37°C to a depth of 16 mm for 1 and 5 minutes, respectively. The temperature was controlled using a water bath machine (GFL, Germany). Immediately after the immersion, the files were rinsed with bidistilled water at 37°C to neutralize the effect of the irrigating solution, dried, and stored in labeled glass containers.

For the purpose of this study, a testing device was made of a stainless-steel block fixed to a main frame. The steel block was machined according to the recommendations of Larsen et al. [25] and Haikel et al. [26] and possessed a simulated root canal, 25 mm in length, with a single curvature of 60° and 5 mm radius that was engraved into the block to a depth of 1.5 mm. The length of the canal was verified by inserting a *K*-file size 15. The steel block was covered with a removable glass plate to help visualize the file in motion, secure the file in the canal, and allow for the easy retrieval of the broken file fragments.

An ultraslow electric motor handpiece (X-Smart; Dentsply Maillefer), with a gear ratio 16 : 1, was attached to the frame and accurately positioned in a reproducible relationship to the steel block such that each file would be oriented in the middle of the artificial canal, perpendicular to the orifice. Care was taken to ensure that the glass cover was not in contact with the file. A lubrication spray (Pegasus Hand Piece Lubrication Spray, UK) was introduced within the canal space to reduce frictional heat.

Cyclic fatigue for all the rotary files was conducted in a water bath machine (GFL, Germany) filled with water maintained at 37°C. Test was done under continuous clockwise rotation at the individual manufacturer's recommended speed for each system. The entire process was video recorded, from commencement of rotation, until file fracture was observed, using a mobile camera. Thereafter, the total number of cycles to fracture (NCF) for each file was calculated by multiplying the time (seconds) to fracture by the number of rotations or cycles per second.

The Shapiro–Wilk test of the NCF of the files showed the data to be normally distributed (W_(90)_ = 0.43, *p*=0.234), necessitating the use of parametric statistics. One-way ANOVA with multiple Bonferroni post hoc test was used to compare the significance of difference among the different groups. The level of confidence for all tests was set at *p* < 0.05. All data were analyzed using the SPSS ver. 25 data processing software (IBM Corp. Armonk, NY, USA).

## 3. Results

The NCF was compared among the different groups tested and subjected to the one-way ANOVA (Table 1). The one-way ANOVA showed significant differences among the different files tested (*F* = 33.152, *p* < 0.001).

It was observed that the files of the OC group had higher NCF than the files of the PTG group regardless of immersion protocol. The multiple Bonferroni tests showed that the PTG-5-min NaOCl had significantly lower NCF than all other groups except for PTG-1-min NaOCl. The PTG-1-min NaOCl group had a higher NCF than the PTG-5-min NaOCl and a lower NCF than the PTG-control group. However, these differences were not statistically significant. The PTG-1-min NaOCl group had a significantly lower NCF than the three protocols of the OC group. The PTG-control group had lower NCF than the files of the OC group. While this difference was statistically significant for the OC-control and OC-1-min NaOCl groups, the difference was not statistically significant when compared to the OC-5-min NaOCl.

The OC-control group had significantly higher NCF than all the other groups in the study. The OC-1-min NaOCl group had a significantly lower NCF when compared to the OC-control group. Although the NCF of the OC-1-min NaOCl was higher than the OC-5-min NaOCl group, the difference was not statistically significant.

## 4. Discussion

The present study compared the cyclic fatigue resistance of two NiTi files, OC with PTG, after immersion in 2.5% NaOCl for one and five minutes at 37°C, followed by cyclic fatigue testing which was also done at 37°C in a water bath. The significance of conducting both the immersion and testing at body temperature was to mimic the clinical condition and thereby obtain dependable data [11, 27]. Care was also taken to immerse only 16 mm from the tip of files in the solution, to avoid galvanic corrosion phenomena [12].

The overall inference obtained from the results of this study is that OC is significantly more resistant to cyclic fatigue failure compared to PTG, thereby refuting the null hypothesis.

Although there have been no prior research done comparing these particular brands of NiTi files for their resistant to cyclic fatigue, several studies have compared OC with other NiTi files, all of which reported that OC files showed a higher cyclic fatigue resistance compared to other tested files [20–24], which is in agreement with the results of the present study.

Heat-treated files demonstrate a higher resistance to fatigue failure than the austenitic and M-wire-based files [16]. PTG and OC files are both heat-treated; however, the latter is based on a C-wire alloy which is a proprietary material, exclusively developed and implemented by Micro Mega, and files are said to have controlled memory and ability to prebend for easier access to the root canal along with elimination of constraints [19].

Cross-sectional geometry and center core area affect the flexibility of the rotary NiTi files [2, 14]. A previous study found that files with high cross-sectional and center core areas are stiffer than files with other designs. Triangular cross sections resulted in increased flexibility than square designs at the same external taper [15]. OC files have a triangular cross section [19], while PTG files have a convex triangular cross section [17]; this increased mass in the center core area of the PTG files because the convex triangular design might explain the reduced fatigue resistance compared with OC files. In a recent study, the core diameter of OC files was measured and found to be very minimal (approximately 48,327 *µ*m^2^) compared to other tested files, which enhances its flexibility and subsequent fatigue resistance [22].

It has also been reported that files with larger taper have less resistance to fatigue failure than files with smaller taper [15]. Although the tip sizes for both tested files, in the present study, are ISO 25, their tapers vary. The taper for PTG/F2 is 0.08 for the apical three millimeters, which progressively decreases until D16 [17], whereas OC files have a constant taper of 0.06 from the tip until D16 [19]. This might additionally justify the increased fatigue resistance of OC size 25 over PTG/F2 in the present study.

Additionally, it was specifically inferred, from the present study, that the cyclic fatigue resistance for both files was significantly decreased after 5 minutes of immersion in NaOCl.

Several studies, with conflicting results, have been conducted to evaluate the effect on files immersed in NaOCl. In a previous study, ProFile and RaCe NiTi files size 25 taper 04 immersed in a heated 5.25% NaOCl solution for 1 or 2 hours showed significant reduction in fatigue resistance by 40% or more [7]. Immersion of Reciproc R25 and WaveOne in 2.5% NaOCl for 5 min decreased the cyclic fatigue resistance significantly (*p* < 0.05) as compared to the control group [10].

Palma et al. [12] concluded in their study that immersion in 3% NaOCl solution for 1 or 5 min decreased the resistance to cyclic fatigue of ProTaper Next, Hyflex CM, and Hyflex EDM, with more significance shown for those manufactured from CM wire. A study in 2018 reported that the cyclic fatigue resistance of PTG files immersed in NaOCl dropped significantly (*p* < 0.05) when compared to those immersed in distilled water. The reduction in the resistance to cyclic fatigue when exposed to NaOCl can be attributed to the induced corrosive areas. It has been stated that active ClO^−^ anions can increase corrosion [28]. Corrosion is sought to create selective removal of surface nickel with micropitting, which makes microstructural defects turn into cracks when submitted to stress, leading to a weakened instrument structure [3].

Although the abovementioned studies are in agreement with the results of the present study, the opposite has also been reported. A study found that static or dynamic immersion in 5% NaOCl for 1 minute or 5 minutes does not significantly reduce the cyclic fatigue resistance of Twisted Files, Revo S SU files, and Mtwo files [3]. Similarly, another study concluded that reciprocating dynamic immersion in 5% NaOCl for 1 or 5 minutes did not reduce significantly the cyclic fatigue resistance of One Shape, Reciproc R25, or WaveOne Primary [9]. Another study was conducted in 2018, which assessed the effects of sodium hypochlorite (NaOCl) immersion and sterilization on the cyclic fatigue resistance of Twisted files and Hyflex CM. They concluded that the cyclic fatigue resistance of the two heat-treated NiTi files tested was not significantly reduced following immersion in NaOCl [13].

The method used in this study for cyclic fatigue was based on a method described by Pruett et al. [29]. This method accurately describes the root canal curvature based on the angle of curvature and radius of curvature, and it provided the files with a suitable simulated root canal with a 60° angle of curvature and 5 mm radius of curvature, measured according to the method of Schneider [30]. The artificial canal wall was flooded with synthetic oil to minimize the friction between the tested files and the simulated canal wall [31]. To remain within a realistic timeframe of clinical practice and considering the mean life of the control instruments, 1 minute and 5 minutes were selected as the contact time of the solution with the file.

The limitations in the study were that both the immersion in sodium hypochlorite and the cyclic fatigue test were done in a static position rather than a dynamic motion. A dynamic motion would mimic the clinical scenario more than a static position.

Within the limitations of this study, it can be concluded that OC files showed significantly more resistance to cyclic fatigue failure compared to PTG under the test conditions. Furthermore, immersion in 2.5% NaOCl for 5 minutes significantly reduced the cyclic fatigue resistance for both the tested files.

## Figures and Tables

**Table 1:** Comparison of mean number of cycles to fracture (NCF) with post hoc Bonferroni comparisons.

Number of cycles to fracture (NCF)	Mean	Standard deviation	95% confidence interval for mean		*F* ^*∗*^	*p*
			Lb	Ub		
PTG-control^a,e^	752.00	165.37	660.42	843.58	33.152	*p* ≤ 0.001
PTG-1-min NaOCl^a,b^	651.00	165.64	559.27	742.73		
PTG-5-min NaOCl^b^	490.33	120.65	423.52	557.14		
OC-control^c^	1325.00	334.70	1139.65	1510.35		
OC-1-min NaOCl^d^	1038.33	204.12	925.30	1151.37		
OC-5-min NaOCl^d,e^	962.33	145.80	881.59	1043.07		

^*∗*^Calculated using one-way ANOVA. ^a,b,c,d,e^Differences in superscript indicate significant (*p* < 0.05) differences between groups.

## Data Availability

The data used to support the findings of this study can be made available upon request to the corresponding author.
